# Instrumental Labor

**Published:** 1878-03-20

**Authors:** G. McDonald


### INSTRUMENTAL LABOR.

(Reported to the Medical Society of Virginia.)

By G. McDonald, M.D.

*Mr. President and Fellows*—The question under consideration is one of the most important of all obstetric questions. For, in those cases of obstructed labor that "try the souls of men" and women, the lives of mothers and children, and the happiness and welfare of families, will depend on the manner in which the accoucheur performs his responsible duties.

I now propose, very briefly, to consider some of the most important operations for the relief of some of the distressing accidents and incidents of parturition; and, first, I hold that every man, who presumes to practice midwifery (especially in the country, where often a consultation is impossible), is bound to know, not only how, in the best manner, to perform all the various operations—not only must he know how and when to operate, but, in any given case, he must be able to decide promptly which operation is applicable.

And first in regard to version. Turning is most frequently required in cases of transverse presentation, accidental or unavoidable hemorrhage, sometimes for slightly contracted pelvis, and occasionally for prolapse of the funis.

I shall not discuss the subject further than to call attention to a mode now practiced in some cases of cephalic version, by external manipulation, applicable only in the early stage of labor, where the waters have not been discharged; and to another mode of performing podalic version by combined external and internal manipulation—that is, by introducing one or two fingers through the os, and thus pushing the head or shoulder, as the case may be, towards the fundus, while the external hand pushes the breech towards the os. These operations are said to be, in suitable cases, entirely practicable, and of easy accomplishment. But, of course, neither of these modes is at all applicable where the waters have been long discharged, and there is rigid tonic contraction of the uterus. Under these distressing circumstances, nothing remains to be done but first to relax the uterus by an anæsthetic, and then patiently and gently, but persistently, introduce the hand, according to the old method, and effect podalic version. But if, on account of rigid contraction of the uterus, and impaction of the presentation in the pelvis, this be impossible, then we have no other means left, except mutilation of the child, either by evisceration and severance of the spine, or decapitation.

And now we turn, with peculiar pleasure, to the use of the forceps, the great conservative instrument of midwifery—conservative of mother and child. That there has been a great revolution in regard to the use of the forceps, we presume is well understood by most, if not all, the members of this society.

We are happy to say that the doctrine, so long taught in the standard works and in the schools, no longer prevails, namely, that the use of the forceps was unjustifiable, unless all hope of natural delivery was at an end, and symptoms of commencing exhaustion were present.

The rule that now prevails, as expressed by another, is, "That so long as nature is able to effect her purpose without prejudice to the constitution of the patient, without danger to the soft parts or to the life of the child, we must let the labor proceed. But as soon as we find the natural efforts are beginning to fail, and after having tried milder means for relaxing the parts or stimulating the uterus to increased action, without result, we are bound, by common sense and common humanity, to relieve the sufferer from her misery, and her child from imminent danger of death, by the timely use of the forceps. Why, may I ask, should we permit a fellow-creature to undergo hours of torture, when we have the means of relief in our hands? Why should the mother be allowed to waste her strength and incur the risk consequent upon long pressure of the head on the soft parts, the tendency to inflammation and sloughing, or the danger of rupture, to say nothing of the increased danger of puerperal fever, with all its dire results?"

That the modern doctrine is not only safer for the mother, but also for the child, is well established by statistics. Among the most remarkable of these are those of Dr. Hamilton, of Falkirk. He delivers one child out of every seven or eight with the forceps, and he thus delivered seven hundred and thirty-one successive children without a single still-birth. When these facts are considered in connection with the fact that, including all cases of labor, one out of every twenty or thirty children is still-born, it will be seen that Dr. Hamilton's experience is unprecedented in obstetric history.

We may now lay down the following propositions:

1. That when the head is low down in the pelvis, and the os uteri fully dilated and retracted over the head, and especially if rotation has taken place, the use of the forceps is a safe operation for mother and child, and so simple that any country doctor ought to be able to perform it with ease and safety.

2. That in a large majority of the cases, where the operation is required, all the above conditions will have occurred.

3. That, the above conditions being present, and the pains being good, but insufficient to cause the expulsion of the child, the use of the forceps is, in the hands of a judicious and skillful operator, safer than the use of any oxytoxic, especially ergot.

4. That, in a majority of cases, the death of the child and injury to the mother (such as sloughing of the vagina, and consequent recto or vesico-vaginal fistula) is caused, not by the use of the forceps, but by the want of them, or too long delay in their use.

5. That, as soon as the physician is satisfied that the strong probability is that the natural powers will be unable to effect delivery (especially if the pains be strong and are not making any impression), there should be interference, and that without regard to the duration of labor—always, provided it be the second stage of labor, and especially if there be great suffering or any danger to mother or child.

6. That in any case where there is greater risk to mother and child by delay, than in using the forceps, they should be resorted to.

7. That the above rules are not intended to justify the unnecessary or bungling use of the forceps.

8. That, in the use of the forceps, after their introduction, and after they are securely locked, an anæsthetic should be administered, provided the operator understands its administration.

And now, sir, we wish it distinctly understood that all we have said relates to the operation, when the head is low in the pelvis, and the os fully dilated and retracted over the head. When the head remains high in the pelvis, and especially if the os be not fully dilated and retracted, the operation is much more formidable, and should not be undertaken except for good reasons, and then with a full appreciation of the difficulty and danger.

We have not undertaken to describe the mode of introducing or handling the forceps, because, on these points, full instruction is given in the standard works. We beg leave, however, to mention that in a recent standard work, we saw the suggestion made, that in all cases, without regard to the position of the head, the forceps should be introduced laterally, thus simplifying the operation.

And now, a few words in relation to that serious operation involving danger to the mother and death to the child—craniotomy:

1. It should never be resorted to if it be possible for a living child to be born without great danger to the mother. Of course, it should never be practiced if the forceps will answer the purpose. The limits to the operation, in some standard works, are said to be from two and three-fourths to three inches antero-posterior diameter, and one and three-fourths inches as the other limit, provided, in the last case, there be good transverse dimensions. That is, I understand the author to mean, that above the limit of two and three-fourths to three inches antero-posterior diameter, it is possible for a living child to be drawn through the pelvis, and that a mutilated child may be extracted through a pelvis of one and three-fourths inches antero-posterior diameter, provided the transverse diameter is good. Hence, the limits of craniotomy would be between the figures above mentioned.

Having determined that the operation is required, the head should be perforated, and then we have choice between cephalotripsy and craniotomy, provided we have a cephalotribe.

Regarding the question of the morality of the operation, where the alternative is between the destruction of the child and the painful and lingering death of both mother and child, we presume there is no question among us. That a certain school of theologians have, in time past, decided that the destruction of the child is a mortal sin, will not, we presume, disturb our consciences, when we know that unless the operation is done, not only will the child inevitably perish, but while we wait for the death of the child, the mother not only suffers untold and unimagined tortures, but there will be imminent danger of her death also.

As I have been so fortunate as to have had but little experience in craniotomy, I leave the subject to those who have been less fortunate.

And now, we have but a word to say in regard to the gravest of all obstetric operations—the dreadful alternative of the Cæsarean section. But dreadful as the necessity is, medical men must be able promptly to decide when it is required, and having so decided, there should be some one in every neighborhood with the requisite courage and skill to perform it. The cases requiring the operation are—

1. When it is impossible to deliver a living child.

2. When it is impossible to deliver a mutilated child without the danger being greater than the Cæsarean operation.

3. The operation ought to be performed with the same precision, care, and skill, and with all the caution necessary in ovariotomy. Under these conditions, and not otherwise, may we hope for success.

4. And, finally, the operation must not be deferred until the case is hopeless, and death must be the result with or without the operation.—*Va. Med. Monthly.*

